# Transcranial magnetic stimulation cortical oscillations and improve cognition in obstructive sleep apnea patients

**DOI:** 10.1002/brb3.2958

**Published:** 2023-03-15

**Authors:** Xiaoxiao Han, Sijie Cai, Hao Gui, Rui Chen

**Affiliations:** ^1^ Department of Respiratory and Critical Care Medicine, Sleep Center, the Second Affiliated Hospital of Soochow University Soochow University Suzhou China; ^2^ Department of Hyperbaric Oxygen The Second People's Hospital of Hefei Hefei Hospital Affiliated to Anhui Medical University Hefei China; ^3^ Department of Pulmonary and Critical Care Medicine Affiliated Kunshan Hospital of Jiangsu University Suzhou China

**Keywords:** cognition, EEG oscillations, OSA, power spectral density, TMS

## Abstract

**Background:**

Transcranial magnetic stimulation (TMS) is a noninvasive tool to improve cognition. Relevant clinical studies are mainly focused on neurological and psychiatric diseases. However, cognition decline and psychiatric disorders are popular in obstructive sleep apnea (OSA) patients. We aimed to investigate the effect of TMS over the left dorsolateral prefrontal cortex (DLPFC) on cognition test performance and to compare the changes in quantitative electroencephalogram (EEG) before and after stimulation for OSA.

**Methods:**

This study recruited 42 OSA patients diagnosed with polysomnography according to American Academy of Sleep Medicine guidelines. TMS (intermittent theta‐burst stimulation paradigm; 2 s on, 8 s off, 600 pulses*3, intermittent 15 min) was performed on the DLPFC. Cambridge Automated Neuropsychological Test Battery was used to assess cognitive performance. EEG oscillations were computed via power spectral density with MATLAB software.

**Results:**

Real‐TMS group displayed a significant improvement in visual memory, sustain attention performance, as well as the outcome of working memory. However, the executive function of latency was changed in both groups. Furthermore, TMS resulted in a significant increase in the relative power spectral density of the theta band and beta band in the parietal, temporal, and anterior regions, respectively.

**Conclusions:**

In summary, our findings indicate that TMS can safely modulate cortical oscillations and improve cognition in OSA patients. In the future, TMS can be utilized as an alternative treatment option to improve cognition in OSA patients.

## Graphical abstract

1. Real‐TMS group displayed a significant improvement in visual memory, sustain attention performance, as well as the outcome of working memory. 2. TMS can be utilized as an alternative treatment option to improve cognition in OSA patients.

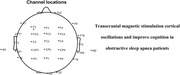

## 1 INTRODUCTION

Obstructive sleep apnea (OSA) is a sleep disorder and a highly prevalent disease. Approximately 26% of adult populations suffer from sleep troubles with OSA (Patil et al., 2019). It is characterized by obvious restriction of airflow (hypopnea and apnea) or episodes of cessation during sleep, which is often associated with detectable arousals on electroencephalography and decreased blood oxygen saturation. OSA is a systemic disease accompanied by many comorbidities. It is associated with all aspects of metabolic syndromes, dyslipidemia, hypertension and obesity, myocardial infarction, congestive heart failure, and stroke (Jordan et al., 2014). All these comorbidities, together with OSA, impair cognitive function, including working memory, executive functions, and attention (Legault et al., 2021). This not only affects the quality of life for patients, but also associates with increased risks of workplace accidents and traffic crashes. As reported, OSA patients have an increased risk of car accidents by 2–10 times (D'Rozario et al., 2013). Numerous experiments have established that OSA is a high‐risk factor for Alzheimer's disease (AD) (Jorge et al., 2019). Continuous positive airway pressure (CPAP) is the gold standard treatment of OSA, and is shown to ameliorate the severity of OSA, while the reversibility of CPAP on the above cognitive impairments is controversial (Bubu et al., 2020; D'Rozario et al., 2022; Patil et al., 2019). Therefore, it is a critical topic to find new methods to improve the cognition of OSA patients.

Transcranial magnetic stimulation (TMS) is a noninvasively interference that is utilized to directly study the physiology and activity of the intact human brain (Nevler & Ash, 2015). TMS modulates the brain excitability synaptic plasticity in the cortical by producing electromagnetic microcurrents. The effects not only focus on subcortical areas to the stimulation site, but also spread into remote and functionally connected corticals (Rabey et al., 2012). TMS has been used to improve cognition in various psychiatric and neurological diseases, including AD (Eliasova et al., 2014; Rabey et al., 2012; Rajji, 2019). The factors affecting the efficacy include stimulus frequency, intensity, stimuli total number, and the interval between stimuli. According to the stimulation frequency, the TMS mode is divided into low frequency (< 1 Hz) and high frequency (> 1 Hz). Intermittent theta‐burst stimulation (iTBS) pattern is a pattern of high‐frequency TMS that prolongs the after‐effects of the cortical changes despite its shorter duration lower stimulus intensity (Vallence et al., 2015). The left dorsolateral prefrontal cortex (DLPFC) is a pivotal hub for network integrations and participates in memory, attention, and executive functions (Xia et al., 2021). DLPFC is one of the most common target stimulation sites in clinical and scientific research.

Cortical oscillations reflect the synchronization of neuronal populations in different frequency bands. The brain function is based on the fundamentals of various frequency bands in oscillation (Henry et al., 2014). Topographic electroencephalogram (EEG) is practicable for implementation in clinical settings as it can assess physiological changes of oscillatory activity in a sensitive and noninvasive way. Moreover, EEG provides time‐sensitive recordings of neural activity. Quantitative analysis of the electroencephalogram (qEEG) has been reported to be effective for identifying small changes that contribute to functional brain tissue alterations (Corsi‐Cabrera et al., 2006). The power spectral analysis is a method of qEEG. The application of qEEG in OSA research has become increasingly popular in recent years. While most of the research has focused on the EEG in sleep, EEG slowing is widely observed (D'Rozario et al., 2013; Liu et al., 2021). By combining EEG and TMS, we can further understand the physiological mechanism underlying the influence of TMS on the excitability of the oscillatory activity. There has previously been no study that investigated the changes in qEEG and cognition performance after TMS stimulation in OSA patients.

In summary, previous studies have established that TMS can produce physiological changes in the brain and the underlying mechanism is related to modulating brain oscillations of different frequency bands. In this pilot study, we aimed to preliminary investigate whether TMS could modulate the cognitive function and alter the oscillatory activity of OSA patients.

## 2 MATERIALS AND METHODS

### 2.1 Subjects

All participants, including 42 OSA patients, were recruited from the Second Affiliated Hospital of Soochow University, and OSA was diagnosed with all‐night polysomnography (PSG) by professional and technical personnel using the American Academy of Sleep Medicine standards. Twenty of them were mild to moderate OSA, and 22 were severe OSA. All participants exhibited decreased cognitive function, such as memory and attention. Participants were randomly divided into the experimental group (i.e., real‐TMS) and the control group (i.e., sham‐TMS).

Inclusion criteria: ages between 30 and 60 years old, right hand, and the education year must be more than 9 years. The exclusion criteria for all subjects included: the presence of psychiatric disorders, history of neurological disorder, and diseases of important organs, such as heart, liver, and kidneys; contraindications to TMS including epilepsy, allergic to conductive paste, metal in the skull, intolerable. Patients must not be treated with ventilator‐assisted ventilation or medications that affect cognition when they were enrolled.

The study was approved by the Ethics Committee of the Second Affiliated Hospital of Soochow University (Ethics Number: JD‐LK‐2021‐062‐01). Written informed consent was derived from the subjects. The study was conducted in accordance with the Declaration of Helsinki.

### 2.2 Clinical measures

#### 2.2.1 Anthropometric and sociodemographic variables

Variables including date of birth, age, sex, education level, body mass index (BMI), history of marriage, and history of smoking and alcohol consumption were collected. PSG data included minimum SaO2%, Oxygen Desaturation Index (ODI), sleep structure (the percentage of sleep time in each stage), and Apnea‐Hypopnea Index (AHI).

#### 2.2.2 Experimental design

**FIGURE 1:**
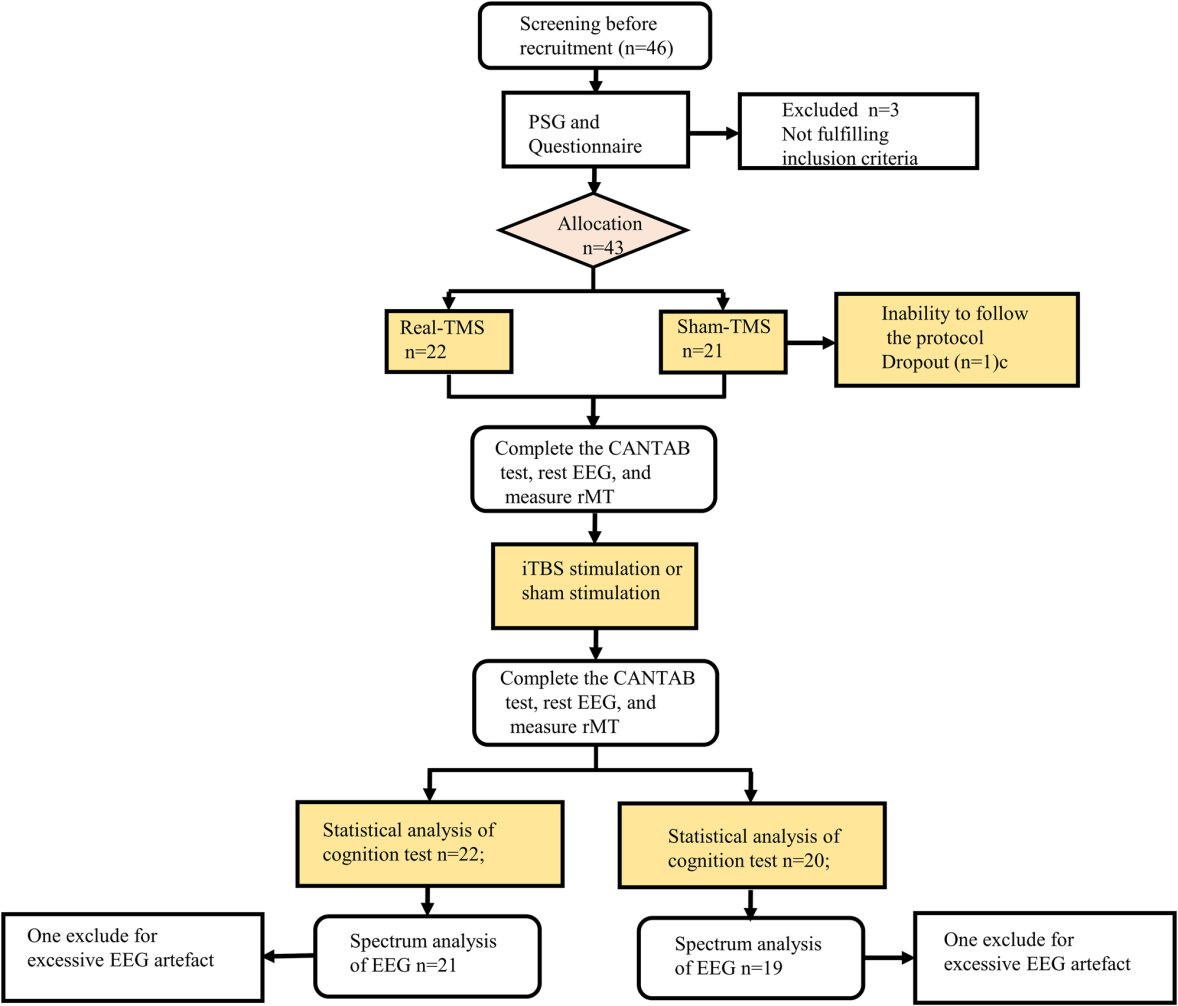
Channel locations. 32 Ag/AgCl electrodes placed according to the 10/20 system and a cephalic (Fpz) location as the ground, referenced to left and right mastoid.

This study utilized a single‐blind, randomized, sham‐controlled design (Figure 1). Participants were randomly assigned to the TMS group (real‐TMS) and control group (sham‐TMS) before experimenting. Subjects were seated in a comfortable armchair in a quiet, dimly light, and sound‐proof room with their body in a relaxed position, and the resting state EEG and resting motor threshold (rMT) were collected. Then, a battery of neuropsychology assessments (i.e., Montreal cognitive assessment [MoCA], Minimum Mental State Examination [MMSE], and the baseline performance of cognition tasks) was required to complete. Subsequently, real‐TMS or sham‐TMS were applied to the participants. Then, EEG and cognition tasks were performed immediately following the TMS intervention.

#### 2.2.3 Questionnaire (neuropsychological battery)

All participants completed the MoCA test, Pittsburgh Sleep Quality Index (PSQI), Epworth, and MMSE.

#### 2.2.4 Cambridge Neuropsychological Test Automated Battery

Cambridge Neuropsychological Test Automated Battery (CANTAB) is currently the most effective and sensitive touchscreen test for measuring cognitive function in the world. Compared with traditional cognitive scales, CANTAB objectively quantifies cognitive function with high specificity and sensitivity in evaluating cognitive impairments. Furthermore, unlike traditional cognitive exams, it is unaffected by speech or culture and minimizes the learning effect. In this study, four tests from CANTAB were conducted at baseline and immediately following stimulation to detect the cognitive functions: I (Pattern Recognition Memory) PRM; ii (Spatial Working Memory) SWM; iii (Rapid Visual Information Processing) RVP; and iv (One Touch Stockings of Cambridge) OTS.

##### Rapid visual information processing

Subjects were asked to find target number sequences (i.e., 2‐4‐6, 3‐5‐7, 4‐6‐8) among a set of pseudorandom numbers, and press the key to record. The results included: total hits (RVPTH), the number of target sequences correctly responded to; probability of hit (RVPPH), calculated from hits/(hits +misses); and RVPA, the signal detection measure of sensitivity to the target, regardless of response tendency. Probabilities and sensitivity were calculated using the Signal Detection Theory measures related to sustained attention.

##### Spatial working memory

Subjects clicked on the squares on the screen to “open” them, find the blue signs, then fill the holes on the right side of the screen with the blue signs. Subjects did not click the same square twice in the same round of searching for the blue mark. As the number of squares increases, the difficulty of this task gradually increases. The outcome measures for the SWM test include: total error (SWMTE), the number of times a box is selected that is certain not to contain a blue token and, therefore, should not have been visited; strategy score (SWMSS), obtained by counting the number of times the subject begins a new search with a different box. A low strategy score indicates good performance, suggesting that there are few repetitive clicks on the squares that have been found in blue.

##### Pattern recognition memory

The screen displayed a series of visual patterns, one at a time. Then, the participant was asked to choose the pattern that just appeared from a novel pattern. The results for the PRM test included: mean latency to correct (PRMMLC), reflecting the speed of reaction; correct percentage (PRMCP), the number of correct responses.

##### One touch stockings

Two patterns were displayed on the screen, and each pattern showed three balls of different colors. The participants had to plan in their minds how many moves the three colored balls would need to be moved to align the positions of the colored balls on the upper and lower monitors. The outcome of OTS included: mean latency to correct (OTSMLC), measured from the appearance of the balls on the screen until the correct box was touched; mean choice to correct (OTSCC), the mean number of choices that the subject made on each problem to make the correct choice.

### 2.3 TMS

Participants included in this study received TMS, with an 80% rMT to the DLPFC. The rMT was defined as the lowest intensity and was estimated before TMS. The mode of TMS was iTBS consisted of 200 busts. The frequency of each bust was 5 Hz applied every 200 ms for 2 s and repeated every 10 s for a total duration of 191 s, each burst consisting of three stimuli pulses delivered at a frequency of 50 Hz, a total of 600 stimuli per session. To achieve a better clinical after effect, three typical iTBS were delivered three times at 15 min with a total of 1800 stimuli (Nettekoven et al., 2014). The sham group used a dedicated placebo coil, which was the same shape as the real coil and produced the same stimulating sound and sensation.

### 2.4 EEG recording and analysis

**FIGURE 2:**
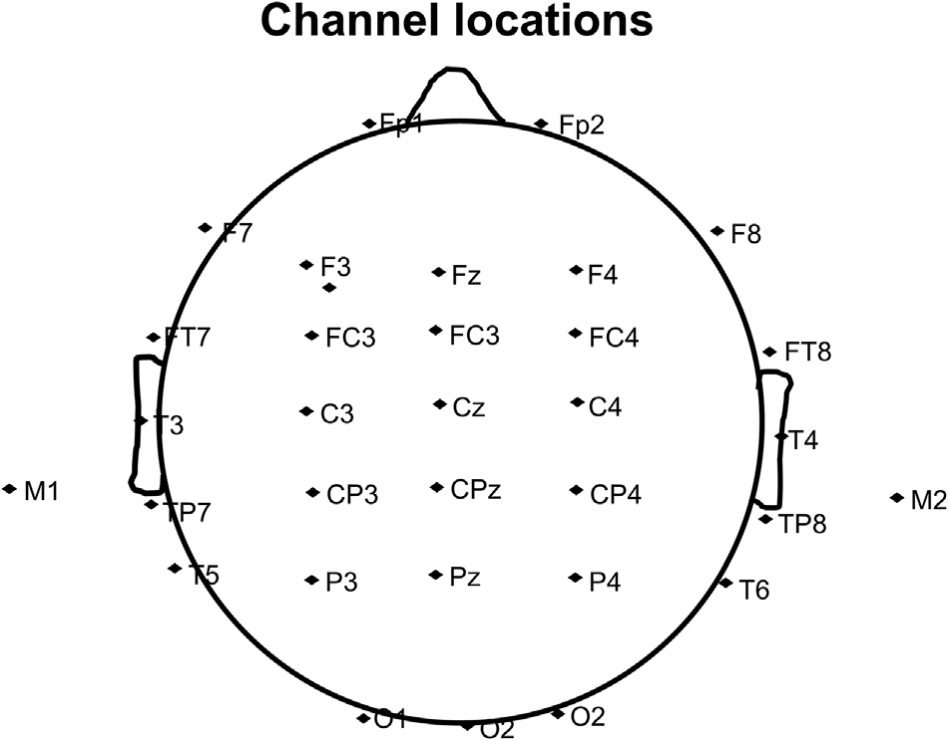
Overview of the experiment design. Of the 46 participants, three subjects did not fulfill the inclusion criteria. The 43 subjects left, were randomly assigned to the real‐TMS group (*n* = 22) and the sham‐TMS group (*n* = 21). In the sham‐TMS group, one was dropped out due to the inability to follow the protocol.

Baseline EEG was collected 10 min per‐TMS treatment, and post‐TMS treatment was collected within 10 min after treatment, each for 10 min. Throughout the monitoring process, the subjects were monitored with electroencephalographic signs of sleepiness tendency. EEG was continuously recorded (bandpass 0.01−100 Hz, sampling rate 1024 Hz) using a Compumedics Grael Amplifier by a set of 32 Ag/AgCl electrodes (Fp1, Fp2, F7, F3, Fz, F4, F8, T3, C3, Cz, C4, T4, T5, P3, Pz, P4, T6, O1, Oz, O2, FT7, FC3, FCZ, FC4, FT, T P7, CP3, CPZ, CP4, TP8, M1, M2) placed according to the 10/20 system and a cephalic (Fpz) location as the ground, referenced to left and right mastoid (Figure 2). The impedance of the whole recording process was lower than 5 kΩ.

To demonstrate the TMS‐induced oscillation changes, spectrum analysis of EEG data was performed with MATLAB software and its signal analysis toolbox (EEGLAB). The EEG data were then bandpass filtered with a 1‐ to 50 Hz finite impulse response filter down sampled to 256 Hz, All EEG data were visually inspected to remove the bad segments (excessively noisy EEG, muscle artifacts), retaining the less drifting EEG. The ocular and prominent muscle artifacts were removed by the combination of automated EEGLAB processes and the independent component analysis. The algebraic average of the electrodes was then rereferenced to the right and left mastoids. MATLAB was also used for further EEG spectrum analysis.

Neural oscillations were quantified based on fast‐Fourier transforms using a Hanning window. Then, the comment power spectrum was found on each lead, and the mean relative power density was computed for each of the following frequency bands: beta (14−29.9 Hz), alpha (8−13.9 Hz), theta (4−7.9 Hz), and delta (1−3.9 Hz). Individual electrodes were aggregated to create an average for the following regions: occipital (O1, Oz, O2), temporal (FT7, FT8, T7, T8, TP7, TP8), parietal (P7, P3, Pz, P4, P8), central (C3, FCz, Cz, CPz, C4), and anterior (F7, F3, Fz, F4, F8).

### 2.5 Statistical analysis

Statistical calculations were performed using SPSS 22.0. For each variable, the Gaussian distribution was evaluated by the Shapiro−Wilk test. Data were expressed as mean ± standard deviation (SD) or median with 25th and 75th percentile.

Distributions of baseline demographic and neuropsychological variables were compared using the independent sample *t*‐test or two‐sample nonparametric Wilcoxon rank‐sum test (Table 1). To objectively quantify and evaluate the short‐term effects of rTMS on cognition performance and qEEG measures, we compared clinical changes per‐ and post‐stimulation of the two groups with paired sample test. All tests were performed as two‐tailed tests.

**TABLE 1:** Baseline characteristics (baseline characteristics of participants)

	Real‐TMS (*n* = 22)	Sham (*n* = 20)	*p*
Age (years)	37.72 (7.06)	39.35 (8.17)	.490
BMI (kg/m^2^)	27.81 (4.00)	26.03 (5.60)	.239
Education age (years)	16.00 (15.75, 17.25)	16.00 (15.00, 16.75)	.458
rMT	40.63 (5.45)	42.80 (6.24)	.238
AHI (per hour)	43.08 (27.84)	39.34 (28.16)	.667
ODI (per hour)	26.35 (18.00, 68.45)	23.85 (7.70, 57.30)	.371
Minimum SaO2 (%)	72.54 (11.30)	73.05 (14.33)	.899
Proportion of SaO2 < 90%	4.45 (1.92, 28.82)	7.50 (0.52, 26.37)	.811
N1 percentage (%)	17.40 (7.27, 25.62)	13.00 (7.65, 24.82)	.850
N2 percentage (%)	50.80 (11.66)	52.31 (11.00)	.670
REM percentage (%)	20.13 (6.41)	19.61 (7.00)	.802
SWS N1 percentage (%)	10.82 (8.56 )	10.97 (7.36)	.953
MoCA	26.00 (25.00, 28.00)	26.50 (24.00, 8.00)	.859
MMSE	30.00 (30.00, 30.00)	30.00 (29.00, 30.00)	.178
PSQI	6.72 (3.26)	6.70 (2.55)	.976
Epworth	9.86 (6.23)	9.15 (4.86)	.684

Abbreviations: AHI, apnea‐hypopnea index; BMI, body mass index; MoCA, Montreal Cognitive Assessment; MMSE, Minimum Mental State Examination; ODI, Oxygen Desaturation Index; PSQI, Pittsburgh Sleep Quality Index; rMT, resting motor threshold.

The association between the clinical features and qEEG measures or cognitive outcomes was determined by exploratory analyses using Pearson's correlation coefficients.

## 3 RESULTS

### 3.1 Demographic and baseline data of the subjects

Out of the 46 participants who were screened, three subjects did not fulfill the inclusion criteria. Among the remaining 43 subjects, 22 were randomly assigned to the real‐TMS group and 21 to the sham‐TMS group. In the sham‐TMS group, one dropped out due to incompliance to follow the protocol. No significant differences were found between the two groups in terms of demographics (age, education level, BMI), PSG data (AHI, ODI, minimum SaO2%), neuropsychological battery (MoCA, MMSE), Epworth sleepiness scale, PSQI, or rMT (*p* > .05) (Table 1).

### 3.2 Effects of iTBS on cognitive performance

The scores of CANTAB tests of the two groups at baseline were comparable. No significant difference was observed between the two groups (*p* > .05) (Table 2).

**TABLE 2:** CANTAB test scores at bassline

Test		Real‐TMS (*n* = 22)	Sham‐TMS (*n* = 20)	*p*
SMM	Strategy	35.00 (30.75, 37.00)	33.00 (27.75, 34.00)	.103
	Time to first response (ms)	1540.62 (9.27, 2267.47)	1403.25 (1010.97, 2175.33)	.481
RVP	A	0.91 ± 0.04	0.92 ± 0.045	.529
	Probability of hit	0.69 ± 0.15	0.72 ± 0.16	.527
	Total hits	18.63 ± 4.23	19.50 ± 4.52	.527
PRM	Percent correct (%)	87.50 (79.16, 91.66)	91.66 (87.50, 95.83)	.122
	Correct latency (ms)	1734.99 (1497.85, 1963.00)	1758.40 (1444.90, 2095.22)	.880
OTS	Choice to correct	1.07 (1.03, 1.26)	1.12 (1.06, 1.20)	.674
	Latency to correct (ms)	10,434.17 (8297.56, 16,907.25)	12,109.22 (8182.81, 15,116.16)	.840

Abbreviations: OTS, one touch stockings; PRM, pattern recognition memory; RVP, rapid visual information processing; SWM, spatial working memory.

**FIGURE 3:**
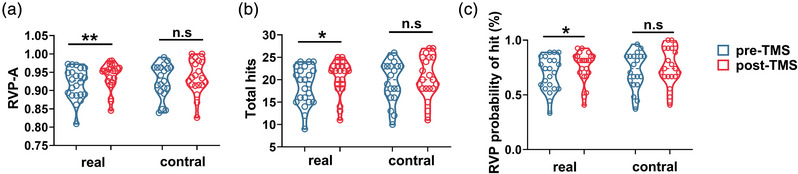
The change of RVP outcome before and after TMS in two groups. There was a significant improvement in the real‐TMS group for RVPA (a), total hits (b), probability of hit score (c) after iTBS, but not in the sham group. * *p* < .05; ** *p* < .01; n.s means *p* > .05.

**FIGURE 4:**
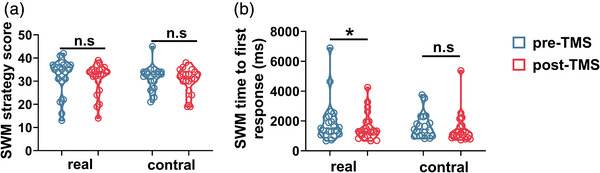
The change of SWM outcome before and after TMS in two groups. The change of the strategy score (a), and the time to first response (b) both groups. * *p* < .05; n.s means *p* > .05.

**FIGURE 5:**
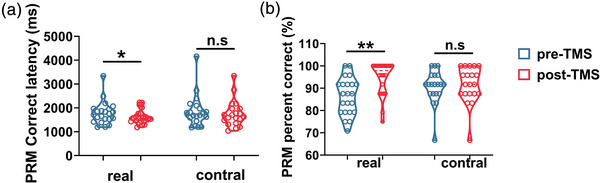
The change of PRM outcome before and after TMS in two groups. Only the real‐group participants showed an improvement in the correct latency (a) and percent correct score (b). * *p* < .05; ** *p* < .01; n.s means *p* > .05.

**FIGURE 6:**
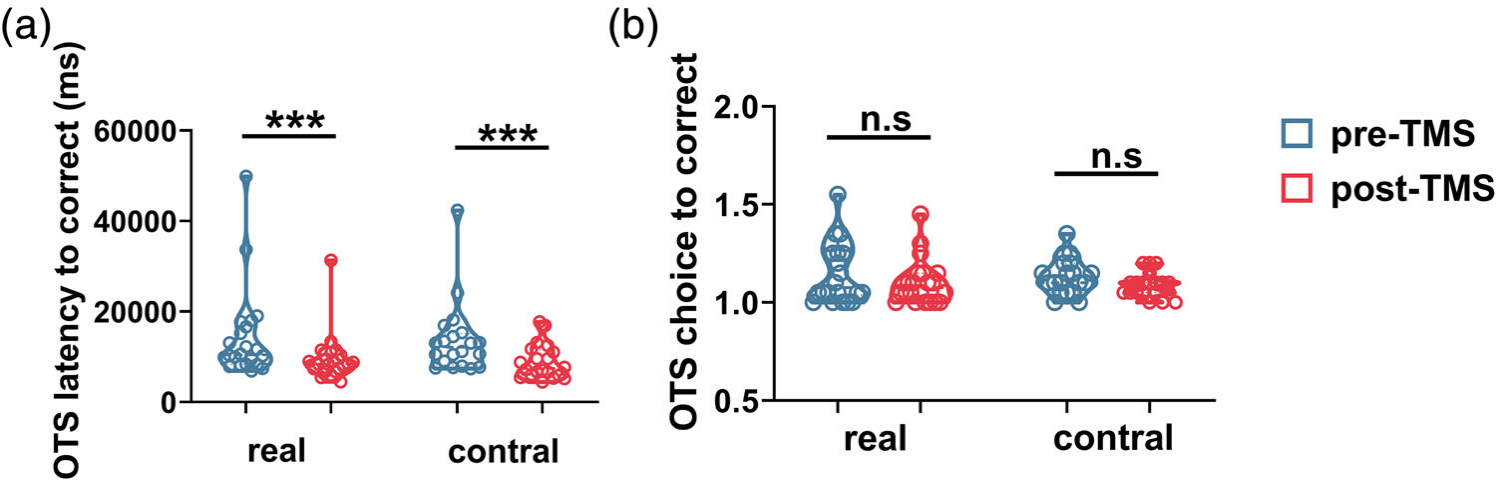
The change of OTS outcome before and after TMS in two groups. In both groups, changes in the latency to correct are statistically significant (a), while neither group's performance improved on the choice to correct (b). *** *p* < .001; n.s means *p* > .05.

Paired‐sample comparisons of the change in CANTAB performance were conducted in two groups (Table 3). In the RVP task, there was a significant improvement in the real‐TMS group for RVPA (0.91 ± 0.04 vs. 0.93 ± 0.036), total hits (18.63 ± 4.23 vs. 20.54 ± 3.82), probability of hit score (18.63 ± 4.23 vs. 20.54 ± 3.826) after iTBS (Figure 3). However, no significant improvement was observed in the sham group. In the SWM, only the real‐TMS group showed significant improvement in the time to first response (real‐TMS: 1540.62 [1109.27, 2267.47] vs. 1301.87 [1024.33, 1959.29]; sham‐TMS: 1403.25 [1010.97, 2175.33] vs. 1221.958 [904.83, 1668.79]), while the change of the strategy score did not meet statistical significance in either group (Figure 4). In the PRM task, the percent correct score (87.50 [79.16, 91.66] vs. 97.91 [87.50, 100.00]) and correct latency (1734.99 [1497.85, 1963.00] vs. 1575.33 [1451.83, 1764.79]) were improved only in the real‐group (Figure 5). Changes on the latency to correct of OTS task in both groups met statistical significance (real‐TMS: 10,434.17 [8297.56, 16,907.25] vs. 8420.70 [6945.45, 10,502.05]; sham‐TMS: 12,109.22 [8182.81, 151,16.16] vs. 7419.80 [5903.97, 107,23.42]). While the choice to correct of OTS did not improve in the real‐TMS group (*p* > .05), a trend of improvement was found in the sham group (1.12 [1.06, 1.20] vs. 1.10 [1.05, 1.13], *p* = .050) (Figure 6).

**TABLE 3:** Change of CANTAB test scores after TMS for two groups

		Real‐TMS (*n* = 22)		
Test		Baseline	Follow‐up	P1
SMM	Strategy	35.00 (30.75, 37.00)	33.50 (29.00, 35.00)	0.262
	Time to first response	1540.62 (9.27, 2267.47)	1301.87 (1024.33, 1959.29)	0.022
RVP	A	0.91 ± 0.04	0.93 ± 0.036	0.009
	Probability of hit	0.69 ± 0.15	0.76 ± 0.14	0.020
	Total hits	18.63 ± 4.23	20.54 ± 3.82	0.020
PRM	Percent to correct (%)	87.50 (79.16, 91.66)	97.91 (87.50, 100.00)	0.001
	Correct latency (ms)	1734.99 (1497.85, 1963.00)	1575.33 (1451.83, 1764.79)	0.036
OTS	Choice to correct	1.07 (1.03, 1.26)	1.07 (1.00, 1.15)	0.119
	Latency to correct (ms)	10,434.17 (8297.56, 16,907.25)	8420.70 (6945.45, 10,502.05)	0.000
		Sham‐TMS (*n* = 20)		
		Baseline	Follow‐up	P2
SMM	Strategy	33.00 (27.75, 34.00)	31.50 (27.00, 33.75)	0.168
	Time to first response	1403.25 (1010.97, 2175.33)	1221.95 (904.83, 1668.79)	0.179
RVP	A	0.92 ± 0.04	0.93 ± 0.04	0.145
	Probability of hit	0.72 ± 0.16	0.76 ± 0.16	0.163
	Total hits	19.50 ± 4.52	20.55 ± 4.57	0.163
PRM	Percent to correct (%)	91.66 (87.50, 95.83)	91.66 (87.50, 98.95)	0.521
	Correct latency (ms)	1758.40 (1444.90, 2095.22)	1664.86 (1384.73, 1967.00)	0.064
OTS	Choice to correct	1.12 (1.06, 1.20)	1.10 (1.05, 1.13)	0.050
	Latency to correct (ms)	12,109.22 (8182.81, 15,116.16)	7419.80 (5903.97, 10,723.42)	0.000

Abbreviations: OTS, one touch stockings; PRM, pattern recognition memory; RVP, rapid visual information processing; SWM, spatial working memory.

### 3.3 Effects of iTBS on power spectral density

EEG data from two of the 42 participants were excluded due to excessive EEG artifacts. The difference (*p* < .05) in power spectral density of brain region between real‐TMS of per‐stimulation and post‐stimulation is presented in Table 4. There was no significant difference in relative power spectral density in the two groups of participants at baseline.

**TABLE 4:** Effect of TMS change on neural oscillations

		Real‐TMS (*n* = 21)			Sham‐TMS (*n* = 19)		
Brain region		Per‐TMS	Post‐TMS	P1	Per‐TMS	Post‐TMS	P2
Anterior	delta	0.60 ± 0.21	0.54 ± 0.19	0.114	0.52 ± 0.16	0.52 ± 0.17	0.952
	theta	0.13 ± 0.05	0.16 ± 0.05	0.065	0.16 ± 0.05	0.16 ± 0.05	0.710
	alpha	0.20 ± 0.17	0.22 ± 0.16	0.471	0.26 ± 0.15	0.25 ± 0.17	0.724
	beta	0.03 ± 0.02	0.04 ± 0.03	0.045	0.03 ± 0.01	0.04 ± 0.02	0.169
Central	delta	0.53 ± 0.22	0.46 ± 0.21	0.078	0.46 ± 0.17	0.47 ± 0.19	0.834
	theta	0.15 ± 0.06	0.18 ± 0.07	0.069	0.16 ± 0.05	0.16 ± 0.06	0.793
	alpha	0.25 ± 0.18	0.27 ± 0.18	0.449	0.32 ± 0.17	0.30 ± 0.19	0.619
	beta	0.04 ± 0.03	0.04 ± 0.04	0.241	0.04 ± 0.02	0.04 ± 0.02	0.327
Parietal	delta	0.54 ± 0.25	0.49 ± 0.22	0.331	0.45 ± 0.19	0.45 ± 0.19	0.998
	theta	0.12 ± 0.05	0.15 ± 0.07	0.028	0.12 ± 0.04	0.14 ± 0.04	0.285
	alpha	0.27 ± 0.21	0.27 ± 0.19	0.903	0.37 ± 0.19	0.34 ± 0.20	0.573
	beta	0.04 ± 0.03	0.04 ± 0.03	0.399	0.04 ± 0.02	0.04 ± 0.02	0.084
Temporal	delta	0.62 ± 0.20	0.53 ± 0.20	0.070	0.57 ± 0.18	0.54 ± 0.18	0.389
	theta	0.11 ± 0.04	0.15 ± 0.05	0.036	0.13 ± 0.04	0.14 ± 0.04	0.338
	alpha	0.17 ± 0.13	0.21 ± 0.13	0.160	0.22 ± 0.13	0.22 ± 0.14	0.993
	beta	0.05 ± 0.05	0.06 ± 0.06	0.267	0.04 ± 0.02	0.05 ± 0.03	0.109
Occipital	delta	0.53 ± 0.24	0.46 ± 0.24	0.136	0.47 ± 0.21	0.49 ± 0.21	0.797
	theta	0.11 ± 0.04	0.14 ± 0.07	0.073	0.12 ± 0.04	0.13 ± 0.04	0.217
	alpha	0.25 ± 0.19	0.25 ± 0.17	0.967	0.31 ± 0.18	0.28 ± 0.17	0.538
	beta	0.05 ± 0.04	0.07 ± 0.08	0.128	0.05 ± 0.03	0.06 ± 0.04	0.536

*Note*: The neural oscillation of each brain region is the average among its electrodes: anterior (F7, F3, Fz, F4, F8), central (C3, FCz, Cz, CPz, C4), parietal (P7, P3, Pz, P4, P8), temporal (FT7, FT8, T7, T8, TP7, TP8), and occipital (O1, Oz, O2).

The change after TMS in the real‐TMS group was observed in the theta band and beta band. Alterations in relative theta band power were in temporal and parietal areas. However, the change in the beta band was limited to the anterior regions.

Contrary to this, no statistically significant change was found in the sham group after TMS in any of the four bands.

## 4 DISCUSSION

OSA is frequently accompanied by neurocognitive impairment, often involving attention, memory, and executive function (Canessa et al., 2018; Olaithe et al., 2018). The prevalence of mild cognitive impairment (MCI) in OSA ranges from 11% to 17%, and the severity of cognitive impairment is not necessarily equivalent to the severity of OSA (Liguori et al., 2020). CPAP treatment can decrease hypoxia degree and improve daytime sleepiness, and is the gold standard treatment of OSA, while the impact of CPAP on cognitive enhancement is modest and controversial (Bubu et al., 2020). Saunamaki et al. (2010) found that after 6 months of CPAP treatment, OSA patients’ visuospatial organizational skills failed to improve. Furthermore, a meta‐analysis was performed to assess the efficacy of CPAP in improving cognitive function in OSA, but failed to demonstrate any significant differences between CPAP and control groups in all domains of cognition function (Patil et al., 2019). EEG is a promising tool for the early detection of cognitive impairment, while the study conducted by D'Rozario et al. (2022) found that the EEG slowing ratio increased in patients after CPAP treatment, suggesting the limitations of CPAP on cognitive improvement and brain damage. Substantial heterogeneity in outcomes and lower adherence to CPAP treatment are additional challenging issues.

In recent years, there are many studies related to the cognition improvement effect of electrical or magnetic stimulation, suggesting that TMS is a promising intervention. TMS can modulate cortical oscillation excitability. These effects can be measured by EEG spectrum, while functional connectivity strength can be measured by EEG or magnetic resonance imaging. Numerous studies, including randomized and nonrandomized controlled studies, have revealed the cognitive promotion effects of TMS on healthy people, MCI, sleep deprivation, and AD (Eliasova et al., 2014; Rabey et al., 2012; Rajji, 2019). For example, Drumond Marra et al. (2015) conducted a randomized controlled trial and found cognition significantly improved in the experimental group compared to the control group after TMS for MCI. Chiara Bagattini et al. found that TMS had add‐on treatment effects in enhancing cognitive for AD patients undergoing cognitive training combined with TMS stimulation, and this effect was affected by the severity of the disease and the education level: AD with less damage or higher educated level showed more benefit. Therefore, intervention should be carried out as soon as possible for patients with cognitive impairment. Furthermore, Li et al. (2022) tried to explore the molecular mechanisms underlying TMS. They found that the excitability of the cerebral cortex changed with statistical significance after one session of TMS stimulation, and proposed that rHF‐TMS mainly regulated glutamatergic neurotransmission. Wang Kai conducted a randomized controlled trial on the effects of high‐frequency repetitive TMS and TBS on cognition to investigate which stimulation mode showed stronger improvement on cognition. The research revealed that after one stimulation, the two groups had different levels of improvement in working memory performance compared to the control group. More importantly, TBS also enhanced executive function (Wu et al., 2021). Prior research on OSA and TMS has mostly focused on the excitability and plasticity of the cortical region. One study applied TMS as a treatment for OSA by stimulating the phrenic nerve to improve airway symptoms (Herrero Babiloni et al., 2018). In light of the previous research findings, we performed the current pilot study to investigate the impact of TBS on the cognition of OSA, and to prepare for future research. In our study, we found improvements in RVP, SWM, and PRM in OSA patients after TMS, that were absent in the control group. This is consistent with the results of previous studies, suggesting that TMS not only improves cognition in neuropsychiatric disorders, but also improves cognition in OSA patients. A previous study demonstrated that a reduction of beta activity was associated with decreased sustained attention and vigilance performance (Muñoz‐Torres et al., 2011). Theta band is a core electrophysiological mechanism in internally directed attention, learning, and memory, and is also essential to executing conflict network (Herweg et al., 2020). In our study, variations in the power spectrum were found in theta and beta bands, increased post‐stimulation compared to pre‐stimulation. The relative power spectrum of the alpha band in the anterior, central, parietal, and temporal regions increased after stimulation, but failed to reach statistical significance. These alterations in EEG oscillations may contribute to enhanced cognitive performance, but more research is needed to investigate further.

These results suggest that the iTBS over DLPDC potentially modulates cognition and modifies the oscillatory activity of OSA patients, providing the framework for additional in‐depth investigation.

### 4.1 Tolerability and safety

iTBS at 80% of the rMT was safe and well‐tolerated. Side effects were mild and transiently prevailing (Lefaucheur et al., 2020). Theoretically, TMS may induce seizures, but the risk is very low. Patients with a history or a family history of epilepsy were excluded from our study. Only two in the real‐TMS group and one in the sham‐TMS group complained of a slight head discomfort which was relieved after half an hour of rest. There were no serious adverse events in either treatment group. Therefore, applying TMS intervention to OSA is safe.

### 4.2 Strengths and limitations

First of all, this is the first randomized controlled study to explore the effect of TMS on OSA patients. Second, we used CANTAB for cognitive assessment, which is an objective and sensitive evaluation measure. However, these neurological assessments are not widely used. In addition, we did not include female subjects and did not group subjects by disease severity. Meanwhile, due to the lack of MRI data, we cannot more intuitively assess the impact of TMS on brain structure. Finally, studies with larger sample sizes are needed to further determine the safety and efficacy of TMS in the long run. At the same time, the effects of different stimulation modes on different cognitive regions also need to be further explored.

## 5 CONCLUSIONS

Our results demonstrate that TMS can influence cerebral cortical activity and improve cognitive performance. TMS is a promising noninvasive therapy for cognitive enhancement in individuals with OSA.

## CONFLICT OF INTEREST STATEMENT

All of the authors declare that they have no conflict of interest.

### PEER REVIEW

The peer review history for this article is available at https://publons.com/publon/10.1002/brb3.2958.

## Data Availability

Data could be obtained upon reasonable request to the corresponding author.

## References

[brb32958-bib-0002] Bubu, O. M. , Andrade, A. G. , Umasabor‐Bubu, O. Q. , Hogan, M. M. , Turner, A. D. , De Leon, M. J. , Ogedegbe, G. , Ayappa, I. , Jean‐Louis, G. , Jackson, M. L. , Varga, A. W. , & Osorio, R. S. (2020). Obstructive sleep apnea, cognition and Alzheimer's disease: A systematic review integrating three decades of multidisciplinary research. Sleep Medicine Reviews, 50, 101250. 10.1016/j.smrv.2019.101250 31881487 PMC7593825

[brb32958-bib-0003] Canessa, N. , Castronovo, V. , Cappa, S. F. , Marelli, S. , Iadanza, A. , Falini, A. , & Ferini‐Strambi, L. (2018). Sleep apnea: Altered brain connectivity underlying a working‐memory challenge. Neuroimaging Clinics, 19, 56–65. 10.1016/j.nicl.2018.03.036 PMC6051941 30035002

[brb32958-bib-0004] Corsi‐Cabrera, M. , Muñoz‐Torres, Z. , Del Río‐Portilla, Y. , & Guevara, M. A. (2006). Power and coherent oscillations distinguish REM sleep, stage 1 and wakefulness. International Journal of Psychophysiology, 60, 59–66. 10.1016/j.ijpsycho.2005.05.004 15996777

[brb32958-bib-0005] D'rozario, A. L. , Hoyos, C. M. , Wong, K. K. H. , Unger, G. , Kim, J. W. , Vakulin, A. , Kao, C.‐H. , Naismith, S. L. , Bartlett, D. J. , & Grunstein, R. R. (2022). Improvements in cognitive function and quantitative sleep EEG in OSA after six months of CPAP treatment. Sleep, 45, *zsac013*. 10.1093/sleep/zsac013 PMC9189957 35029691

[brb32958-bib-0006] D'rozario, A. L. , Kim, J. W. , Wong, K. K. H. , Bartlett, D. J. , Marshall, N. S. , Dijk, D. ‐J. , Robinson, P. A. , & Grunstein, R. R. (2013). A new EEG biomarker of neurobehavioural impairment and sleepiness in sleep apnea patients and controls during extended wakefulness. Clinical Neurophysiology, 124, 1605–1614. 10.1016/j.clinph.2013.02.022 23562656

[brb32958-bib-0007] Eliasova, I. , Anderkova, L. , Marecek, R. , & Rektorova, I. (2014). Non‐invasive brain stimulation of the right inferior frontal gyrus may improve attention in early Alzheimer's disease: A pilot study. Journal of the Neurological Sciences, 346, 318–322. 10.1016/j.jns.2014.08.036 25216556

[brb32958-bib-0008] Henry, M. J. , Herrmann, B. , & Obleser, J. (2014). Entrained neural oscillations in multiple frequency bands comodulate behavior. Proceedings of the National Academy of Sciences of the United States of America, 111, 14935–14940. 10.1073/pnas.1408741111 25267634 PMC4205645

[brb32958-bib-0009] Herrero Babiloni, A. , De Beaumont, L. , & Lavigne, G. J. (2018). Transcranial magnetic stimulation: Potential use in obstructive sleep apnea and sleep bruxism. Sleep Medicine Clinics, 13, 571–582. 10.1016/j.jsmc.2018.07.002 30396450

[brb32958-bib-0010] Herweg, N. A. , Solomon, E. A. , & Kahana, M. J. (2020). Theta oscillations in human memory. Trends in Cognitive Sciences, 24, 208–227. 10.1016/j.tics.2019.12.006 32029359 PMC8310425

[brb32958-bib-0011] Jordan, A. S. , Mcsharry, D. G. , & Malhotra, A. (2014). Adult obstructive sleep apnoea. Lancet, 383, 736–747. 10.1016/S0140-6736(13)60734-5 23910433 PMC3909558

[brb32958-bib-0012] Jorge, C. , Benítez, I. , Torres, G. , Dakterzada, F. , Minguez, O. , Huerto, R. , Pujol, M. , Carnes, A. , Gaeta, A. M. , Dalmases, M. , Gibert, A. , Sanchez De La Torres, M. , Barbé, F. , & Piñol‐Ripoll, G. (2019). The Stop‐Bang and Berlin questionnaires to identify obstructive sleep apnoea in Alzheimer's disease patients. Sleep Medicine, 57, 15–20. 10.1016/j.sleep.2019.01.033 30897451

[brb32958-bib-0013] Lefaucheur, J. ‐P. , Aleman, A. , Baeken, C. , Benninger, D. H. , Brunelin, J. , Di Lazzaro, V. , Filipović, S. R. , Grefkes, C. , Hasan, A. , Hummel, F. C. , Jääskeläinen, S. K. , Langguth, B. , Leocani, L. , Londero, A. , Nardone, R. , Nguyen, J.‐P. , Nyffeler, T. , Oliveira‐Maia, A. J. , Oliviero, A. , … Ziemann, U. (2020). Evidence‐based guidelines on the therapeutic use of repetitive transcranial magnetic stimulation (rTMS): An update (2014–2018). Clinical Neurophysiology, 131, 474–528. 10.1016/j.clinph.2019.11.002 31901449

[brb32958-bib-0014] Legault, J. , Thompson, C. , Martineau‐Dussault, M. ‐È. , André, C. , Baril, A. ‐A. , Martinez Villar, G. , Carrier, J. , & Gosselin, N. (2021). Obstructive sleep apnea and cognitive decline: A review of potential vulnerability and protective factors. Brain Sciences, 11, 706. 10.3390/brainsci11060706 34071739 PMC8226698

[brb32958-bib-0015] Li, C. ‐T. , Juan, C. ‐H. , Lin, H. ‐C. , Cheng, C. ‐M. , Wu, H. ‐T. , Yang, B. ‐H. , Tsai, S. ‐J. , Su, T. ‐P. , & Fitzgerald, P. B. (2022). Cortical excitatory and inhibitory correlates of the fronto‐limbic circuit in major depression and differential effects of left frontal brain stimulation in a randomized sham‐controlled trial. Journal of Affective Disorders, 311, 364–370. 10.1016/j.jad.2022.05.107 35618168

[brb32958-bib-0016] Liguori, C. , Maestri, M. , Spanetta, M. , Placidi, F. , Bonanni, E. , Mercuri, N. B. , & Guarnieri, B. (2020). Sleep‐disordered breathing and the risk of Alzheimer's disease. Sleep Medicine Reviews, 55, 101375. 10.1016/j.smrv.2020.101375 33022476

[brb32958-bib-0017] Liu, S. , Shen, J. , Li, Y. , Wang, J. , Wang, J. , Xu, J. , Wang, Q. , & Chen, R. (2021). EEG power spectral analysis of abnormal cortical activations during REM/NREM sleep in obstructive sleep apnea. Frontiers in Neurology, 12, 643855. 10.3389/fneur.2021.643855 33716946 PMC7953149

[brb32958-bib-0018] Marra, D. , L, H. , Myczkowski, M. L. , Memoria, C. M. , Arnaut, D. , Ribeiro, P. L. , Mansur, C. G. S. , Alberto, R. L. , Bellini, B. B. , Fernandes da Silva, A. A. , Tortella, G. , Ciampi de Andrade, D. , Teixeira, M. J. , Forlenza, O. V. , & Marcolin, M. A. (2015). Transcranial magnetic stimulation to address mild cognitive impairment in the elderly: A randomized controlled study. Behavioural Neurology, 2015, 287843. 26160997 10.1155/2015/287843 PMC4487699

[brb32958-bib-0019] Muñoz‐Torres, Z. , del Río‐Portilla, Y. , & Corsi‐Cabrera, M. (2011). Diazepam‐induced changes in EEG oscillations during performance of a sustained attention task. Journal of Clinical Neurophysiology, 28, 394–399. 21811130 10.1097/WNP.0b013e318227323a

[brb32958-bib-0020] Nettekoven, C. , Volz, L. J. , Kutscha, M. , Pool, E. ‐M. , Rehme, A. K. , Eickhoff, S. B. , Fink, G. R. , & Grefkes, C. (2014). Dose‐dependent effects of theta burst rTMS on cortical excitability and resting‐state connectivity of the human motor system. Journal of Neuroscience, 34, 6849–6859. 10.1523/JNEUROSCI.4993-13.2014 24828639 PMC4019799

[brb32958-bib-0021] Nevler, N. , & Ash, E. L. (2015). TMS as a tool for examining cognitive processing. Current Neurology and Neuroscience Reports, 15, 52. 10.1007/s11910-015-0575-8 26092315

[brb32958-bib-0022] Olaithe, M. , Bucks, R. S. , Hillman, D. R. , & Eastwood, P. R. (2018). Cognitive deficits in obstructive sleep apnea: Insights from a meta‐review and comparison with deficits observed in COPD, insomnia, and sleep deprivation. Sleep Medicine Reviews, 38, 39–49. 10.1016/j.smrv.2017.03.005 28760549

[brb32958-bib-0023] Patil, S. P. , Ayappa, I. A. , Caples, S. M. , Kimoff, R. J. , Patel, S. R. , & Harrod, C. G. (2019). Treatment of adult obstructive sleep apnea with positive airway pressure: An American Academy of Sleep Medicine systematic review, meta‐analysis, and grade assessment. Journal of Clinical Sleep Medicine, 15, 301–334. 10.5664/jcsm.7638 30736888 PMC6374080

[brb32958-bib-0024] Rabey, J. M. , Dobronevsky, E. , Aichenbaum, S. , Gonen, O. , Marton, R. G. , & Khaigrekht, M. (2012). Repetitive transcranial magnetic stimulation combined with cognitive training is a safe and effective modality for the treatment of Alzheimer's disease: A randomized, double‐blind study. Journal of Neural Transmission, 120, 813–819. 10.1007/s00702-012-0902-z 23076723

[brb32958-bib-0025] Rajji, T. K. (2019). Transcranial magnetic and electrical stimulation in Alzheimer's disease and mild cognitive impairment: A review of randomized controlled trials. Clinical Pharmacology & Therapeutics, 106, 776–780. 10.1002/cpt.1574 31321766

[brb32958-bib-0026] Saunamäki, T. , Himanen, S.‐L. , Polo, O. , & Jehkonen, M. (2010). Executive dysfunction and learning effect after continuous positive airway pressure treatment in patients with obstructive sleep apnea syndrome. European Neurology, 63, 215–220. 10.1159/000278301 20215753

[brb32958-bib-0027] Vallence, A.‐M. , Goldsworthy, M. R. , Hodyl, N. A. , Semmler, J. G. , Pitcher, J. B. , & Ridding, M. C. (2015). Inter‐ and intra‐subject variability of motor cortex plasticity following continuous theta‐burst stimulation. Neuroscience, 304, 266–278. 10.1016/j.neuroscience.2015.07.043 26208843

[brb32958-bib-0028] Wu, X. , Wang, L. , Geng, Z. , Wei, L. , Yan, Y. , Xie, C. , Chen, X. , Ji, G.‐J. , Tian, Y. , & Wang, K. (2021). Improved cognitive promotion through accelerated magnetic stimulation. eNeuro, 8, 10.1523/ENEURO.0392-20.2020 PMC7901150 33452108

[brb32958-bib-0029] Xia, X. , Li, Y. , Wang, Y. , Xia, J. , Lin, Y. , Zhang, X. , Liu, Y. , & Zhang, J. (2021). Functional role of dorsolateral prefrontal cortex in the modulation of cognitive bias. Psychophysiology, 58, e13894. 10.1111/psyp.13894 34227119

